# Estimating the survival advantage based on telomere length and serum biomarkers of aging

**DOI:** 10.1186/s12967-017-1267-8

**Published:** 2017-08-01

**Authors:** Yilin Zhao, Shijun Li, Hui Liu

**Affiliations:** College of Medical Laboratory, Dalian Medical University, Dalian, 116044 China

**Keywords:** Aging, Albumin, Telomere length, Survival advantage

## Abstract

**Background:**

This study aimed to establish a model that estimates the survival advantage at the molecular level based on telomere length and serum biomarkers of aging, to explore clinical significance.

**Methods:**

The study consisted of 100 healthy subjects and 40 type 2 diabetes mellitus patients, 20–90 years of age. Saliva telomere relative length (LnTL) was measured by the quantitative real-time polymerase chain reaction and the serum biochemical parameters, including albumin (ALB), total proteins, total cholesterol, triglycerides, and some enzyme parameters were detected by a biochemical analyzer. The Z values were transformed from mean values and standard deviations to estimate the survival advantage. A normal reference range (95% confidence interval) was set to the comprehensive advantage of the Z values (Z_s_) to evaluate the comprehensive survival advantage.

**Results:**

The Z values of serum ALB and saliva LnTL could be used to estimate the survival advantage, and effectively distinguish between the aging and nonaging individuals. The Z_s_ was greater than 1.64 in the normal reference range, and type 2 diabetes mellitus patients had lower survival advantages compared to those of the control group (*p* < 0.05).

**Conclusions:**

Our two-dimensional model system using ALB and LnTL was valid and may have potential applications for evaluating the aging status at the molecular level, and for the observation of disease characteristics.

## Background

The survival advantage is the indicator that reflects the ability to resist environment and oxidative stress and other adverse conditions that are essential for health assessments to predict the survival advantage [1, 2]. The survival advantage model we studied was defined as the stronger survival potential ability of an individual among her/his peers, showing a longer life expectancy. At present, only using aging markers to evaluate healthy body status is not comprehensive [3], and therefore the survival advantage of this type of study is decreased. We evaluated the survival advantage based on the molecular markers of aging to establish a survival advantage model.

The breakdown of tissues and organs by diseases or aging was first revealed on a molecular level, and involves the long-term accumulation of DNA damage leading to cellular aging and death [4]. This could affect survival ability; therefore, health assessments may be related to early molecular changes that could be used to quantitate the health problems of aging. Because the aging process is dynamic, aging biomarkers are often incorporated into a model of survival advantage [5, 6].

At present, most of the biomarkers of aging include changes in DNA, RNA, proteins, and body fluids, which can reflect the degree of aging [7, 8]. Telomere length has been recognized as an excellent marker of aging [9]. The telomere is a complex composed of specific proteins and telomeric DNA, and is located at the two terminal ends of eukaryotic chromosomes. Telomeres play an important role in maintaining chromosome stability [10, 11]. However, hereditary traits, various oxidative stresses, and the environment reduce telomerase activity in the cortex and gray matter, myocardial cells, germ cells, and almost all somatic cells. The telomere DNA gradually shortens, eventually leading to cell senescence and death. The measurement of telomere length can be used to evaluate the aging status of the organism at the molecular level, and could further reflect genetic changes [12, 13].

In individuals older than 75 years, telomere length increased with age when compared to individuals less than 75 years old [14, 15]. Regarding these findings, we hypothesized that in the same age range, individuals with longer telomeres versus shorter telomeres did not die at the same time, and when compared with the subjects in the long telomere length group, those individuals in the short telomere length group had a higher probability of mortality. Therefore, longer telomeres showed a significant survival advantage in elderly people. However, in addition to telomere length, we screened for additional serum markers of aging to establish a more robust model that included multiple parameters.

Changes in serum proteins, total cholesterol, triglycerides, enzymes, and other biochemical components are observed with aging [16–18]. The physiological functions of individuals at increasing ages can be measured as abnormal cellular responses to environmental stimuli or stress. Senescent cells show a decline in their physiological function as reflected in abnormal metabolic products that can be measured in the blood [12, 19]. Even in the absence of a disease, older individuals still exhibit great differences in the concentrations and the basic structures of proteins compared with that of younger individuals [7, 20].

With aging, serum lipid breakdown is reduced, cholic acid levels are decreased, and increased total cholesterol content in the liver is observed [21]. The physiological changes caused by aging also cause a functional decline of organs [22]. In addition, the age-affected enzymes in the serum could cause abnormal metabolism, and changes in biochemical components in the blood could indirectly reflect the aging status [23]. These biochemical indicators could be used to quickly and conveniently detect changes with aging, and may provide for more accurate and objective information.

We screened representative markers of aging, and with age, the probability of death was uniform. When compared with other aging markers, telomere length reflected the individual survival advantage, and specific biomarkers of aging were more likely to reflect the overall population aging condition [1, 9]. However, because the parameters were different, their combined use estimated a survival advantage on the molecular level. The main purpose of this study was to establish a survival advantage model, to determine if telomere length together with serum markers of aging at the molecular level could be used to evaluate the clinical significance of aging.

Type 2 diabetes mellitus is a degenerative disease associated with aging [24]. It mostly occurs in the elderly population, and samples are easy to obtain. Notably, diabetes mellitus is easy to diagnose and the diagnostic parameters are objective and accurate. In this study, type 2 diabetes was used to verify the two-dimensional survival advantage model that we have established.

## Methods

### Subjects

The study was approved by the Institutional Ethics Committee of Dalian Medical University and all subjects agreed to participant in the experiment. From July 2016 to August 2016, 100 healthy volunteers and 40 volunteers with type 2 diabetes mellitus patients were enrolled at the Second Affiliated Hospital of Dalian Medical University (Dalian, China). The inclusion criteria of healthy subjects were: no medication or recent diseases, and females not in their menstrual cycle. Type 2 diabetes mellitus patients were included according to these diagnostic criteria: (1) glycated hemoglobin A1c ≥ 6.5%; (2) fasting plasma glucose ≥ 7.0 mmol/L; or (3) blood glucose at 2 h in the oral glucose tolerance test ≥11.1 mmol/L. The exclusion criteria were: diabetes mellitus patients with complications of heart disease or liver cancer; individuals with a chronic disease that would affect the collection of saliva and telomere length measurement, such as smokers, drinkers, subjects suffering from an oral disease, pregnant women, or those with mental illnesses. This study conformed to the tenets of the Declaration of Helsinki regarding the use of human subjects in research.

### Saliva and serum collection

The saliva of all subjects were collected using specialized salivary collection tubes without any additives (Salivette^®^; Sarstedt, Nümbrecht, Germany) in the morning between 07:00 am and 08:00 am, in a quiet and calm environment. Before the saliva collection, the subjects were asked to avoid drinking water, brushing their teeth, smoking, or chewing food or gum for 30 min. They were advised to then chew on a cotton swab for approximately 45 s; the swab was then placed in the saliva collection tube. The entire collection tube with the swab was centrifuged at 1000×*g* for 2 min, resulting in approximately 1 mL of collected supernatant. After fasting for at least 8 h, a peripheral blood sample was collected, then the sample was centrifuged at 1500×*g* for 10 min, and the supernatant was frozen at −20 °C until further analysis. Saliva and serum were collected from the same subjects at 24 h.

### DNA extraction and telomere length assay

Salivary DNA samples were extracted using a DNA extraction kit (Omega Biotek, Norcross, GA, USA). Two hundred microliters of supernatant from the saliva was used for the extraction of genomic DNA. A spectrophotometer (Thermo Fisher, Waltham, MA, USA) was used to measure the concentration and purity of the DNA that was then stored in a freezer at −20 °C.

Telomere relative length was measured using the fluorescence quantitative polymerase chain reaction (PCR) method of Cawthon [25] to calculate the ratio of the telomere gene to a single copy gene (T/S), and the T/S was proportional to the relative length of the telomere, therefore, T/S was used to express the relative length of the telomere obtained from the saliva.

The upstream primer sequence of the telomere gene was as follows:

5ʹ-CGGTTTGTTTGGGTTTGGGTTTGGGTTTGGGTTTGGGTT-3ʹ.

The downstream primer sequence of telomere gene was as follows:

5ʹ-GGCTTGCCTTACCCTTACCCTTACCCTTACCCTTACCCT-3ʹ.

The telomere gene product fragment was 76 base pairs (bp). The upstream primer sequence of the single gene (*36B4*) was as follows: 5ʹ-CAGCAAGTGGGAAGGTGTAATCC-3ʹ; and the downstream primer sequence of the single gene (*36B4*) was as follows:

5ʹ-CCCATTCTATCATCAACGGGTACAA-3ʹ.

The *36B4* gene product fragment was 63 bp.

All quantitative (q) PCR were performed on the Thermal Cycler Dice Real Time System Single and Lite (TaKaRa, Kyoto, Japan). We used Cawthon’s qPCR method with modifications that input 5 ng DNA [25], and the experimental conditions were 40 cycles, a pre-denaturation temperature of 95 °C, for 30 s; a denaturation temperature of 95 °C, for 15 s, and an annealing temperature of 60 °C for 1 min. Each sample contained three parallel samples, and the average value was taken to calculate T/S. The formula was as follows:

$$ \Delta {\text{Ct}} = {\text{Ct}}_{\text{TEL}} - {\text{Ct}}_{36B4} ,\;{\text{relative}}\;{\text{T/S}}\;({\text{TL}}) = 2^{{ - [({\text{CtTel}} - {\text{Ct}}36B4) - ({\text{CtTel}} - {\text{CtControl}})]}} ,\;{\text{then}}\;{\text{TL}} = 2^{{ - \Delta \Delta {\text{Ct}}}} . $$

### Biochemical parameter assays

All serum sample assays were performed on the automatic biochemical analyzer (Hitachi 7600-110; Hitachi, Tokyo, Japan). The parameters included albumin (ALB), total protein (TP), total cholesterol (TC), triglycerides (TG), alanine aminotransferase (ALT), aspartate aminotransferase (AST), alkaline phosphatase (ALP), glutamyl transpeptidase (GGT), and lactate dehydrogenase (LDH). The indoor environment was controlled, and all the parameters were detected on the same day with the entire determination completed within 2 h.

## Statistical analysis and model establishment

The relative length of the salivary telomeres gave a skewed distribution; therefore, the data were transferred into a natural logarithm and analyzed for the saliva telomere relative length (LnTL). The healthy subjects were divided into groups in a 10-year age range, and the serum biochemical parameters were expressed as medians. The relationship between the biomarkers and age was analyzed by curve regression and scatter plot analyses. Nonparametric tests were used to analyze the variations of different groups. The mean value and standard deviation of each index in all healthy subjects were calculated, and the Z value was calculated as:

Albumin (ALB), total proteins (TP), and LnTL: ${\text{Z}} = ({\bar{\text{X}}} - {\text{X}})/{\text{S}}$ [26, 27].

TC, TG, AST, ALT, ALP, GGT, and LDH: ${\text{Z}} = ({\text{X}} - {\bar{\text{X}}})/{\text{S}} .$

$$ {\text{Z}}_{\text{s}} = [{\text{Z}}\;({\text{LnTL}}) + {\text{Z}}({\text{ALB}})]/2 $$

The Z value was used to establish the survival advantage model, and the *χ* ^*2*^ test was used to analyze the difference between the groups. All statistical analyses used SPSS software (SPSS, Chicago, IL, USA), and a value of p < 0.05 (bilateral) was considered as statistically significant.

## Results

**Fig. 1:**
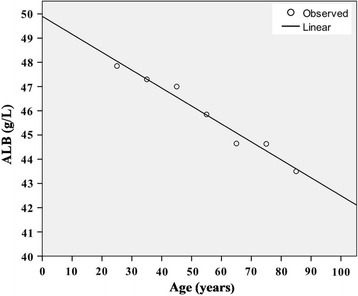
Linear relationship between ALB concentration and age. *p* < 0.001, R^2^ = 0.968. *ALB* albumin

**Fig. 2:**
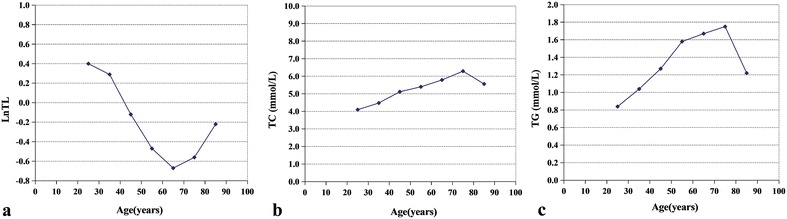
Linear range of observation of the LnTL, TC, and TG, with age. **a** LnTL (the natural logarithm of telomere length), **b** TC (total cholesterol), and **c** TG (triglycerides) with age.* LnTL* r = −0.750, p = 0.052; *TC* r = 0.893, p = 0.007; *TG* r = 0.643, p = 0.119

The saliva LnTL and serum biochemical parameters including ALB, TP, TC, TG, ALT, AST, ALP, GGT, and LDH were analyzed among the different age groups. The results are listed in Table 1. The ALB concentration decreased with age and showed a good correlation with age, (R^2^ = 0.968, *p* < 0.001) (Fig. 1). The LnTL gradually decreased with age, but in the group >65 years of age, there was a reverse rise; similarly, the TC and TG increased gradually before 75 years of age, but decreased in the >75 years of age group, as is shown in Fig. 2.

**Table 1:** Distributions of LnTLs and biochemical parameters in the different age groups

Group (years)	LnTL	ALB (g/L)	TP (g/L)	TC (mmol/L)	TG (mmol/L)	ALT (U/L)	AST (U/L)	ALP (U/L)	GGT (U/L)	LDH (U/L)
20–29	0.40	47.85	73.75	4.10	0.84	6.00	14.00	62.00	14.00	140.00
30–39	0.29	47.30	74.45	4.48	1.04	10.50	18.00	70.00	18.50	143.50
40–49	−0.12	47.00	74.50	5.12	1.27	10.00	16.00	59.00	21.00	156.00
50–59	−0.47	45.85	75.10	5.40	1.58	12.00	21.00	70.00	28.50	157.50
60–69	−0.67	44.65	70.50	5.79	1.67	7.50	17.00	78.50	20.00	178.00
70–79	−0.56	44.64	74.40	6.29	1.75	9.00	17.00	81.50	23.00	172.50
80–89	−0.22	43.50	71.50	5.56	1.22	8.00	19.00	74.00	19.00	177.00
N	98	100	100	100	100	100	100	100	100	100
p	0.677	<0.001	0.446	<0.001	0.009	0.102	0.002	0.051	0.014	<0.001

All parameters are represented by as median

*LnTL* the natural logarithm of telomere length, *ALB* albumin, *TP* total proteins, *TG* triglycerides, *TC* total cholesterol, *ALT* alanine amino transferase, *AST* aspartate amino transferase, *ALP* alkaline phosphatase, *GGT* gamma glutamyl transferase, *LDH* lactate dehydrogenase

**Fig. 3:**
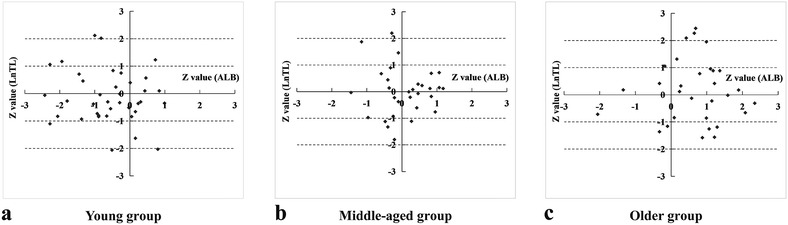
Distributions of **a** the young group, **b** the middle-aged group, and **c** the older age group in the two-dimensional model. The *x-axis* shows the Z value of albumin (ALB) and the *y-axis* shows the value of LnTL (the natural logarithm of telomere length). There was significant difference between the constituent ratios of each group (*p* = 0.004)

Because ALB had a good correlation and the LnTL reflected the survival advantage on a molecular level, both were used to develop our model. Using the Z values of ALB as the x-axis, and LnTL as the y-axis, the I quadrant represented the Z value of both LnTL and ALB (>0); the II quadrant represented the Z value of LnTL (>0) and the Z value of ALB (<0); the III quadrant represented the Z value of both LnTL and ALB (<0); and the IV quadrant represented the Z value of LnTL (<0) and the Z value of ALB (>0). Analyses of the young age group (n = 40; age 20–39 years), the middle-aged group (n = 30; age 40–59 years), and the older-aged group (n = 30; age 60–90 years) in the four quadrants of the distribution and the percentages, are shown in Fig. 3.

There was no significant difference in the distribution of the four quadrants in the diabetic patient group and the control group (*p* = 0.413) (Table 2). However, there were significant differences in the constituent ratio of each age group (*p* = 0.004), as shown in Fig. 3.

The Z_s_ was calculated as follows:

$$ {\text{Z}}_{\text{s}} = [{\text{Z}}\;({\text{LnTL}}) + {\text{Z}}({\text{ALB}})]/2, $$

so when Z_s_ is larger than 1.64, we set a 95% reference interval (single tail). The analyses of the differences of Z_s_ in the diabetic group and control group are shown in Table 3, and were significant (*p* = 0.026).

**Table 2:** Distribution of diabetes and control subjects in the four quadrants

Group	Age (${\bar{\text{x}}}$)	I (%)	II (%)	III (%)	IV (%)	*χ* ^*2*^	*p*
Diabetes	65.19	25.0	10.0	27.5	37.5	2.862	0.413
Control	63.12	35	16.7	18.3	30.0		

*I* represents the Z value of LnTL and ALB both >0; *II* represents the Z value of LnTL >0 and the Z value of ALB <0; *III* represents the Z value of LnTL <0 and the Z value of ALB <0; and *IV* represents the Z value of LnTL <0, and the Z value of ALB >0. All abbreviations are the same as in Table 1

**Table 3:** Comparisons of the percentage between the diabetes and control groups using cutoff Z_s_ values (1.64)

Group	Age (${\bar{\text{x}}}$)	Z_s_ ≤ 1.64 (%)	Z_s_ > 1.64 (%)	*χ* ^*2*^	*p*
Diabetes	65.19	90.5	9.5	5.948	0.026
Control	63.12	100.0	0.0		

The 95% confidence interval was Z > 1.64 (single-tail)

## Discussion

In this study, the preliminary screening included ALB, TC, and TG from nine parameters due to three parameters representing relationships with aging. ALB showed a good linear relationship with age, indicating an aging indicator. We found that the TC and TG had a reverse turning point in the elderly group [28], indicating that the two indicators showed survival advantage indicators similar to telomere length. However, the inflection point was obviously greater for telomere length compared with the above two parameters. Therefore, the final parameter of the survival advantage model was the relative length of the telomere.

For elderly people, longer telomeres may be selective for relatively healthy people, or the elderly having shorter telomeres may have died, and therefore it was not possible to obtain a sample. Likewise, the healthy elderly with a stronger survival and longer telomere length could reflect an organismic survival advantage [1, 9, 14]. It is necessary to establish a new system to evaluate the survival advantages of peers. The Z value of LnTL was relatively smaller than ALB indicating a survival advantage. The two parameters of ALB and LnTL changed independently. Therefore, after standardization, these two parameters were used to establish the two-dimensional evaluation system.

Z value was used to standardize the different parameters. For comparable Z values of ALB and LnTL, we used the standardization to establish a two-dimensional system, when the Z value of LnTL was smaller than ALB (IV quadrant); indicating a survival advantage. Theoretically, the score was equivalent to zero, but individuals with different Z values may not reach the theoretical value. The I quadrant indicated that some individuals were biologically aging and therefore had a poorer survival advantage; the II quadrant indicated that some individuals were not biologically aging and had a poorer survival advantage; the III quadrant indicated that some individuals were not biologically aging and therefore had a stronger survival advantage; and, the IV quadrant indicated that some individuals were biologically aging and had a stronger survival advantage compared to the peers. Therefore, the distribution ratio of individuals among each group in the four quadrants could predict the characteristics of aging.

Our results showed that the II and the IV quadrants represented individuals with poorer and stronger survive advantages compared to the peers, respectively. The younger group and older group mainly distributed in quadrants III and I, respectively, and they were not evenly distributed in the four quadrants, while the middle-aged group was almost evenly distributed in the four quadrants. This demonstrated that the system was effective.

Although our two-dimensional analysis showed no significant difference between the diabetic group and the control group, a greater degree of aging was found in diabetes patients using the comprehensive analysis. Perhaps the chronic diseases did not cause severe enough damage to cause mortality at the molecular level [29]. This finding could also be a characteristic of diabetes. In the future, other diseases such as cancer or heart disease should be studied using the two-dimensional system.

## Conclusion

Our two-dimensional model system using ALB and LnTL was valid and may have potential applications in the evaluation of aging status at the molecular level for the observation of disease characteristics.

## Abbreviations

LnTL: the natural logarithm of telomere length
ALB: albumin
TP: total proteins
TG: triglycerides
TC: total cholesterol
ALT: alanine amino transferase
AST: aspartate amino transferase
ALP: alkaline phosphatase
GGT: gamma glutamyl transferase
LDH: lactate dehydrogenase
